# The Integrated Palliative Outcome Scale (IPOS): A tool for assessing needs and shaping individualized care plans in hospice settings

**DOI:** 10.1017/S1478951526102818

**Published:** 2026-06-04

**Authors:** Erika Iacona, Michela Bondì, Lucia Ronconi, Michela Rigon, Alessia Casati, Ines Testoni

**Affiliations:** 1 Department of Philosophy, Sociology, Pedagogy and Applied Psychology (FISPPA), University of Padova, Padova, PD, Italy; 2 Hospice Paolo VI, Fondazione Opera Immacolata Concezione OIC, Padova, Italy; 3 Department of Biomedical Sciences, University of Padova, Padova, PD, Italy; 4 IT and Statistical Services, Multifunctional Pole of Psychology, University of Padova, Padova, Italy; 5 Department of Political Science, Law and International Studies, University of Padova, Padova, Italy

**Keywords:** Integrated Palliative Outcome Scale, palliative care, individualized care plan, hospice, mixed-method research

## Abstract

**Objectives:**

This mixed-method study examined whether the Integrated Palliative Outcome Scale (IPOS) can support the identification of palliative care needs and inform individualized care planning in hospice settings.

**Methods:**

Thirty-eight terminally ill patients admitted to a hospice in Northern Italy completed the IPOS. Quantitative analyses described the frequency and intensity of physical, psychological, relational, spiritual, and practical needs at the first administration and, where available, compared scores across 2 administrations using non-parametric tests. Spearman correlations were used to explore associations between awareness of diagnosis/prognosis and symptom burden. In parallel, semi-structured interviews explored the subjective meaning of “being at peace with oneself”; responses were examined through thematic content analysis.

**Results:**

IPOS administration highlighted frequent needs related to constipation, oral discomfort, weakness, drowsiness, anxiety, and concerns about family members. Awareness of diagnosis and prognosis were positively correlated. Anxiety and not feeling at peace showed negative associations with awareness, particularly awareness of prognosis. Across administrations, most physical symptoms remained stable, while anxiety and depressive feelings increased. Qualitative findings showed that inner peace was mainly associated with calmness, satisfaction with life, relational fulfilment, not having harmed others, and acceptance of one’s condition.

**Significance of results:**

The integration of IPOS into routine hospice care may help multidisciplinary teams identify patients’ evolving needs and translate them into more responsive individualized care plans. Combining structured IPOS scores with patient narratives can also make existential and psychosocial concerns more visible in clinical decision-making.

## Introduction

Palliative care, as defined by the World Health Organization, aims to improve the quality of life of patients and their families facing life-threatening illness through the prevention and relief of suffering. This approach marks a shift from to cure to care, placing the patient at the center of care pathways and shared decision-making processes (World Health Organization 2023). The WHO’s emphasis on placing the patient at the center of care aligns with the recognition of patient agency as a fundamental component of ethical and effective palliative care. This perspective resonates with the principles set out in the Oviedo Convention, which affirms the primacy of individual autonomy, informed consent, and the right to participate in decisions regarding one’s own health (Council of Europe 1997). In Italy, the principles highlighted by the WHO and the Council of Europe find concrete expression in national legislation. Law No. 38/2010 (Law No. 38/2010 2010) guarantees access to palliative care and pain management as a human right, while Law No. 219/2017 (Law No. 219/2017 2017) affirms the patient’s right to informed consent and advance directives, thus reinforcing the centrality of autonomy and self-determination in medical care. Both Law No. 38/2010 and Law No. 219/2017 reflect the principles promoted by the WHO and the Council of Europe, particularly by endorsing shared care planning between healthcare professionals and patients. This collaborative approach marks a decisive shift away from medical paternalism, fostering mutual trust and reinforcing the patient’s role as an active agent in decisions regarding their own care. Within this framework, it becomes essential to define individualized care plans developed through a relationship of mutual trust between the patient and the healthcare team (Edwards and Elwyn 2009; Sudore et al. 2017; Testoni et al. 2020). Several international tools are available to support individualized care planning by systematically collecting data on patients’ clinical status, values, and preferences. Several tools have been developed over time to support structured, patient-centered decision-making in palliative care. The Support Team Assessment Schedule (STAS), introduced in 1986, was one of the earliest tools designed to evaluate a wide range of clinical and psychosocial problems in palliative settings (Collins et al. 2015). The Edmonton Symptom Assessment System (ESAS), developed in 1989, focuses on the self-reported intensity of common symptoms experienced by palliative patients, such as pain, fatigue, and anxiety (Moro et al. 2006). The Palliative Care Outcome Scale (POS; Hearn and Higginson 1999) is one of the most widely used instruments, providing a structured foundation for informed, person-centered decision-making in the context of shared care by combining patient and staff perspectives on physical, emotional, and existential dimensions of suffering.

In the Italian healthcare system, the Piano Assistenziale Individualizzato (Individualised Care Plan – ICP) serves as the main framework for personalized and multidisciplinary care planning. It is a structured tool developed collaboratively by the healthcare team, in dialogue with the patient and, when appropriate, their family, with the aim of addressing clinical, psychological, social, and spiritual needs in an integrated and dynamic manner (Mercer et al. 2015). The ICP is typically structured into 4 key phases – observation, planning, delivery, and evaluation – and involves the active participation of the patient, family members, and caregivers throughout the process, with the aim of fostering empowerment and shared responsibility in care (Entwistle and Watt 2013). Since STAS, ESAS, and POS lay the foundation for more structured, needs-based, and responsive care planning, to support ICP such instruments have long been employed to ensure systematic assessment of patients’ needs in palliative settings. Building on the strengths of these earlier instruments, the Integrated Palliative Outcome Scale (IPOS) was developed as a more comprehensive tool to assess not only physical symptoms but also the psychological, emotional, spiritual, and practical needs of patients.

IPOS is part of the Outcome Assessment and Complexity Collaborative Suite of Measures, developed by the Cicely Saunders Institute – Palliative and Supportive Care Group at King’s College London, and has been validated across various clinical settings, including in Italy (Murtagh et al. 2014; Schildmann et al. 2016; Veronese et al. 2019), supporting its integration into routine practice across diverse cultural contexts. Distinct from POS, IPOS begins with an open-ended question that invites patients to report the most important problems or concerns they have experienced over the past 3 or 7 days, depending on the version used. This patient-led approach encourages individuals to articulate their priorities, reinforcing their central role in the care process and promoting autonomy and shared decision-making. The domains explored by IPOS are as follows (Veronese et al. 2019).

Assessment of physical needs (the 10 most common physical symptoms in palliative care, with the option to add up to 3 additional symptoms identified by the patient); Assessment of psychological, relational, existential, and spiritual aspects; Assessment of needs related to communication, information, and practical or social concerns. IPOS includes 17 items (plus 3 optional symptoms) covering a broad range of domains, and offers 3 additional blank fields in the physical symptoms section to allow for personalized input on issues not listed among the predefined options. Each item is rated on a 4-point scale, and the tool can be completed by patients, caregivers, or healthcare professionals, ensuring high adaptability across care settings. By combining standardized symptom tracking with open, narrative-based input, IPOS provides a structured yet flexible foundation for developing individualized care plans that truly reflect the values, preferences, and evolving needs of people receiving palliative care.

The primary aim of this study was to identify and quantify the main needs and concerns of terminally ill patients using the IPOS, while monitoring their evolution over time. This approach was intended to uncover specific care requirements and to inform more targeted and individualized support strategies. The specific objectives were: to provide a realistic and comprehensive assessment of the overall condition of each patient; to explore whether IPOS could serve as a suitable tool to support healthcare professionals in completing the ICP, and to evaluate its effectiveness in facilitating personalized care planning.

## Methods

### Design and setting

This study used a mixed-method design combining repeated IPOS administrations with a qualitative exploration of patients’ lived experience in a hospice setting in Northern Italy.

### Participants

A total of 38 terminally ill patients took part in the study. All participants were responsive, without any cognitive impairment, and able to provide informed consent. The sample included 14 women and 24 men, aged between 53 and 93 years (mean age: 75.37; SD = 9.81). Of these, 33 were Italian nationals and 5 were foreign nationals with sufficient proficiency in the Italian language to participate effectively. The most frequently recorded diagnosis was cancer. Regarding illness awareness, 24 participants were fully aware of their diagnosis, 1 was partially aware, and 13 had no awareness. In contrast, awareness of prognosis was markedly lower: 9 participants were fully aware, 4 were partially aware, and 25 were unaware of their prognosis.

Participant characteristics are summarized in Table 1.

**Table 1.** Participants Table 1 long description.

Variable	Range; mean (SD)/N (%)
Age	53–93; 75.37 (9.81)
Gender	
Female	14 (37%)
Male	24 (63%)
Nationality	
Italian	33 (87%)
Other	5 (13%)
Origin	
Hospital	24 (63%)
Home	14 (37%)
Type of pathology	
Carcinoma	34 (89.5%)
Myeloma	1 (3%)
Leukemia	1 (3%)
Lymphoma	1 (3%)
Other pathology	1 (3%)
Psychiatric pathology	
No	32 (84%)
Yes	6 (16%)
Days of hospitalization at Time 1	2–17; 5.39 (4.61)
Days of hospitalization at Time 2	5–31; 11.23 (6.82)
Days of hospitalization at Death	4–67; 24.05 (14.76)
Awareness of diagnosis	
Low	13 (34%)
Medium	1 (3%)
High	24 (63%)
Awareness of prognosis	
Low	25 (66%)
Medium	4 (10.5%)
High	9 (24%)

### Procedure and measure

Data collected through the IPOS were shared with the multidisciplinary care team and used to develop an ICP tailored to the specific needs identified for each patient, while also taking into account the evolution of their clinical condition. The first administration was completed 5 days after hospice admission. Initially, the second administration was planned after 7 days; however, it soon became clear that, except for a few clinically stable patients, this interval was too long. Many patients deteriorated to such an extent that they were no longer able to complete the questionnaire a second time. Therefore, the second administration was moved to 3 days after the first.

### Quantitative analysis

The statistical analysis of quantitative data was performed using SPSS software. For each IPOS item at Time 1, the frequency of the minimum score (0 = Not at all) and maximum score (4 = Always), as well as the mean and standard deviation, were calculated. Given the ordinal nature of IPOS items, non-parametric tests were employed. Correlations between symptom burden and patient awareness were analyzed using Spearman’s rho coefficient. The comparison between the 2 IPOS administrations was carried out using the Wilcoxon signed-rank test. The level of statistical significance was set at *p* < 0.05.

### Qualitative analysis

In the qualitative phase, IPOS was also used as a conversational guide to explore the subjective meaning and emotional weight patients attributed to their sense of peace with themselves. This phase involved a semi-structured interview lasting approximately 30 minutes, conducted at the time of IPOS administration. During these interviews, patients were invited to elaborate on their main concerns and problems, enabling a deeper understanding of their lived experience beyond the numerical scores. A thematic content analysis was conducted to identify the most recurrent concepts emerging from participants’ responses. Following a bottom-up approach, categories were inductively derived from the narratives, allowing themes to emerge directly from the data (Testoni et al. 2021). The procedure followed the 6-phase framework proposed by Braun and Clarke (Braun and Clarke 2006): initial familiarization with the data, generation of preliminary codes, identification of themes, review and refinement of themes, search for alternative explanations, and final report writing.

### Ethical considerations

The study was conducted in accordance with the APA Ethical Principles of Psychologists and Code of Conduct and the principles of the Declaration of Helsinki. Participants were fully informed about the aims of the research and the analytical procedures involved. Informed consent was obtained prior to participation: individuals were asked for permission to audio-record the interviews, to transcribe their responses, and to analyze the resulting data. Participation was voluntary, and only those who provided written and signed consent were included in the study. Anonymity was ensured by assigning pseudonyms to all participants and by slightly modifying quotations to avoid any risk of identification.

The study protocol was approved by the Ethics Committee for Research Involving Human Participants at the University of Padua (approval no. 342-a).

## Results

### Quantitative results

In the first administration, several symptoms showed low frequency, particularly vomiting, nausea, pain, poor communication with clinicians, and not feeling at peace. Only a few symptoms – such as family anxiety, constipation, and oral problems – were reported with higher frequency (see Table 2). A positive correlation was found between awareness of diagnosis and awareness of prognosis (rho = .48, *p* = .002), which allowed for the creation of a composite score (“total awareness”) used to explore associations with symptom burden.

**Table 2.** Results for each item of IPOS at Time 1 and their correlation with awareness of diagnosis and prognosis (*N* = 38) Table 2 long description.

IPOS	0–1 *N* (%)	2 *N* (%)	3–4 *N* (%)	Diagnosis awareness	Prognosis awareness	Total awareness
Symptoms in physical sphere						
Pain	33 (87%)	2 (5%)	3 (8%)	.01	.03	.01
Shortness of breath	24 (63%)	6 (16%)	8 (21%)	.00	.23	.12
Weakness	14 (37%)	7 (18%)	17 (45%)	.09	.19	.16
Nausea	33 (87%)	1 (3%)	4 (10%)	−.17	.00	−.10
Vomiting	35 (92%)	0 (0%)	3 (8%)	−.28	−.24	−.30
Poor appetite	20 (53%)	8 (21%)	10 (26%)	.11	−.06	.03
Constipation (N = 35)	6 (17%)	5 (14%)	24 (69%)	−.12	.11	−.01
Oral problems	12 (32%)	4 (10%)	22 (58%)	−.20	−.17	−.22
Drowsiness	17 (45%)	6 (16%)	15 (39%)	−.12	.05	−.05
Mobilization problems	16 (42%)	6 (16%)	16 (42%)	.19	.21	.23
Symptoms in psychological sphere						
Anxiety (*N* = 36)	19 (53%)	3 (8%)	14 (39%)	−.09	−.41*	−.30
Family anxiety (*N* = 36)	6 (17%)	4 (11%)	26 (72%)	−.14	−.25	−.24
Depressive feeling (*N* = 36)	22 (61%)	2 (6%)	12 (33%)	−.09	−.22	−.19
Symptoms in spiritual sphere						
Feeling at peace – reverse (*N* = 36)	28 (78%)	2 (6%)	6 (17%)	−.32	−.35*	−.38*
Symptoms in relational sphere						
Well sharing with family, friends – reverse (*N* = 36)	21 (58%)	10 (28%)	5 (14%)	−.09	−.25	−.20
Sufficient information by physicians (clinicians) – reverse (*N* = 35)	30 (86%)	1 (3%)	4 (11%)	−.11	−.28	−.23
Practical problems (psycho, social) (*N* = 26)	18 (69%)	2 (8%)	6 (23%)	−.11	−.05	−.09

*Note*: Spearman rho coefficient.

* *p* < .05.

Two symptoms were found to be associated with patient awareness: anxiety and not feeling at peace. Patient anxiety showed a negative correlation with total awareness (rho = – .30, *p* = .075) and with awareness of prognosis (rho = – .41, *p* = .014), but not with awareness of diagnosis (rho = – .09, *p* = .588). Similarly, not feeling at peace was negatively correlated with total awareness (rho = – .39, *p* = .024), as well as with awareness of diagnosis (rho = – .35, *p* = .039) and awareness of prognosis (rho = – .32, *p* = .059).

The distribution of IPOS items at the first administration and their correlations with awareness are reported in Table 2. Changes between the 2 administrations are reported in Table 3.

**Table 3.** Comparison of results for each item of IPOS in the 2 administrations (*N* = 26) Table 3 long description.

IPOS	Time 1 mean (SD)	Time 2 mean (SD)	Wilcoxon *z*	*p*
Symptoms in physical sphere				
Pain	0.35 (0.63)	0.62 (1.17)	−0.75	.454
Shortness of breath	0.85 (1.16)	0.81 (1.33)	−0.18	.857
Weakness	1.73 (1.48)	2.00 (1.65)	−0.90	.366
Nausea	0.50 (1.21)	0.31 (0.88)	−0.92	.357
Vomiting	0.31 (1.09)	0.46 (1.14)	−0.82	.414
Poor appetite	1.15 (1.29)	0.92 (1.23)	−0.96	.337
Constipation	2.85 (1.25)	3.08 (1.29)	−1.23	.210
Oral problems	2.15 (1.54)	2.19 (1.70)	−0.03	.974
Drowsiness	1.77 (1.56)	2.15 (1.40)	−1.34	.182
Mobilization problems	1.54 (1.53)	1.54 (1.33)	−0.13	.894
Symptoms in psychological sphere				
Anxiety	1.52 (1.56)	2.08 (1.69)	−2.01*	.044
Family anxiety	2.68 (1.21)	2.96 (1.02)	−1.17	.083
Depressive feeling	1.08 (1.50)	1.54 (1.61)	−1.93*	.054
Symptoms in spiritual sphere				
Feeling at peace – reverse	0.36 (0.81)	0.31 (0.93)	−0.37	.713
Symptoms in relational sphere				
Well sharing with family, friends – reverse	1.00 (1.12)	0.73 (1.37)	−0.76	.445
Sufficient information by physicians (clinicians) – reverse	0.60 (1.32)	0.80 (1.00)	−1.10	.272
Practical problems (psycho, social)	0.75 (1.25)	1.12 (1.51)	−1.33	.184

*Note:*

* *p* < .05.

Physical symptoms remained stable over time, while only symptoms within the psychological domain showed a significant change between the first and second IPOS administrations. In particular, an increase in anxiety and depressive feelings was observed (see Table 3).

### Qualitative results

The number of participants who responded to the open-ended question decreased from 35 at the first administration to 24 at the second administration. A content analysis was conducted on patients’ responses to the question: “What does being at peace with yourself mean to you?,” which was asked at both time points. Approximately one quarter of participants associated inner peace with a sense of calmness or tranquillity. For example, Jacopo, a 79-year-old Italian man with end-stage lung cancer, stated: “Being calm and being satisfied.” Similarly, Cristina, aged 83 and diagnosed with pancreatic cancer, said: “I feel serene, I’ve lived my life,” while Ludovica, 71 years old with metastatic lung adenocarcinoma, affirmed: “I feel good with myself.”

Another 20% of participants linked being at peace with having achieved personal and relational fulfilment, especially in the family and professional domains. Luigi, aged 87 and diagnosed with terminal bladder cancer, reflected: “I am at peace with my wife too. I have a very united family and have had many satisfactions.” Davide, 83, with a diagnosis of lung cancer, emphasized the importance of having consciously shaped his own life: “I’m satisfied with my life because I chose it. I’ve always thought about others: when my children and grandchildren are well, I’m well too.”

A further 6 out of 35 participants referred to the quality of their relationships with others as essential to feeling at peace. Gabriele, an 80-year-old man with terminal gallbladder cancer, explained: “I’ve never hurt anyone, I’ve always helped others. I will never complain about what I’ve done.” Similarly, Davide stated: “I am 100% sure that I’ve never harmed anyone. I’ve never envied anyone.”

For some, inner peace was closely associated with acceptance of their condition. Franco, 93, affected by advanced renal cancer with bone metastases, shared: “You either accept it or you don’t. Behind everything there’s another side – otherwise, there’s nothing.” Giulio even expressed curiosity about what lies beyond death: “I face everything with an open mind, no problems. Dying? Let’s see what happens – let’s dive in!”

At the second administration, the themes of social connectedness, satisfaction, and tranquility continued to be central. Giuseppe, 74, with a diagnosis of lung cancer, commented: “I’m calm, I did everything I could do.” Domenico, an 80-year-old man with colon cancer, who expressed both serenity and acceptance, said: “I’m calm. I know I can’t make big plans.”

Again at the second administration, some participants identified satisfaction with their life as a key element of inner peace. Ludovica stated: “I’m afraid of dying, but I’m satisfied with what I’ve done.”

## Discussion

The use of the IPOS enabled a multidimensional understanding of patients’ perceptions across the physical, psychological, relational, and spiritual domains. Higher total IPOS scores reflected a greater burden of palliative care needs, allowing the care team to tailor interventions accordingly. To minimize social desirability bias and response distortion, questionnaires were not completed in the presence of family or friends, but were instead administered in the presence of a researcher, who also facilitated in-depth conversations on the issues raised. From the quantitative data, several distressing symptoms emerged as particularly burdensome for terminally ill patients. These included constipation, oral discomfort, anxiety, concern for family members, weakness, and drowsiness. Consistent with the existing literature, constipation – linked to tumor involvement and high-dose opioid therapy – affects between 40% and 90% of patients with advanced cancer (Larkin et al. 2018). This highlights the importance of the care team’s capacity to both prevent and manage constipation, using oral or rectal laxatives, in order to alleviate discomfort and maintain patients’ quality of life. Oral discomfort was also prominent, affecting communication, swallowing, taste, and increasing the risk of oral candidiasis. The dryness often caused by opioids and benzodiazepines remains difficult to treat pharmacologically; current approaches rely on non-pharmacological strategies such as oral hydration and the use of lozenges (Fleming et al. 2020).

Psychological symptoms such as anxiety and worry were also prevalent. This is in line with research showing that approximately 10% of cancer patients experience clinically relevant anxiety, while up to 20% report symptoms of depression (Pitman et al. 2018). Acceptance of one’s condition has been shown to reduce anxiety (Die Trill 2013), while trait anxiety can predispose individuals to more intense psychological distress. Psychosocial interventions are thus essential to help patients process their condition realistically and identify sources of meaning and continuity in their lives. Narrative approaches, in particular, offer patients an opportunity to reconstruct their life story, reaffirm their identity, and feel seen beyond their illness (Zmijewski et al. 2022). The presence of an attentive listener reinforces patients’ sense of dignity and value, providing psychological comfort that pharmacological treatment alone cannot offer.

Moreover, physical fatigue and drowsiness significantly impact quality of life. As reported in other studies, an average of 49% of patients with cancer experience fatigue or lack of energy (Al Maqbali et al. 2021), and in the last 1–4 weeks of life these symptoms may become overwhelming (Hosokawa et al. 2022). Their management, therefore, must be prioritized alongside pain and symptom control. An increase in anxiety from the first to the second IPOS administration may be interpreted in 2 ways. On the one hand, patients who were not aware of their prognosis may experience internal conflict between their bodily sensations and the optimistic information received from others (e.g., “You’ll be home soon”). On the other hand, patients who were aware of their terminal condition may feel increasing distress as the days pass, knowing that death is approaching and out of their control (Kredentser and Chochinov 2020).

While anxiety and depressive feelings increased over time, other symptoms – such as pain, nausea, and vomiting – remained relatively stable. This clinical stability, despite disease progression, suggests that symptom management was timely and effective, reflecting high-quality palliative care provision (Facco et al. 2017).

The qualitative component of the study explored the often-overlooked spiritual domain, revealing its centrality in how patients made sense of their experience. In palliative care, addressing only physical symptoms is insufficient. Patients’ values, preferences, and existential concerns must be acknowledged as integral components of care (Testoni et al. 2016). Empathy and compassion thus become essential tools, promoting dignity and relational presence (Sinclair et al. 2017). Asking patients what it meant to be at peace with themselves opened up a space for existential reflection, reaffirming the personhood and subjectivity of each individual. These narratives challenge reductionist views of the patient as merely the sum of their disease, and instead affirm their status as whole persons who deserve skilled, attentive, and humane care. Through the qualitative question, it was possible to gather narratives that revealed inner dimensions not detectable through standardized instruments. Highlighting how the pursuit of a sense of calm, the perception of having achieved one’s personal and relational goals, and the acceptance of one’s condition are key elements in attaining a feeling of inner peace. These reflections highlighted the presence of profound needs related to the search for meaning, personal reconciliation, and the processing of one’s condition. Integrating these elements allowed for a more nuanced interpretation of the quantitative responses, bringing to light the complexity of the patient’s lived experience.

In line with the World Health Organization’s (World Health Organization 2023) recognition of spiritual well-being as one of the core dimensions of health (Chen et al. 2018), the study confirmed the importance of acknowledging inner needs as an integral part of quality of life.

## Limitations and future research

However, the study presents some limitations. Several items required explanation to ensure full comprehension, especially among older patients. The unpredictable progression of the illness occasionally prevented a second administration at Time 1 (T1), and despite instructions to refer to the previous 3 or 7 days, some participants responded based on how they felt at the time of the interview. Flexibility in administration timing was also necessary, meaning that the standardized intervals were not always followed. Additionally, the study sample was relatively small, with a predominance of older, male, Italian participants, limiting the generalizability of findings.

Future research should aim to involve a larger and more diverse sample in terms of age, gender, and cultural background. Furthermore, we recommend the systematic integration of IPOS into routine clinical practice and its inclusion in the electronic medical record. This would enhance information sharing within the multidisciplinary team and support the development of more holistic and individualized care pathways.

## Conclusions

This study highlights the value of the IPOS as a key instrument for promoting truly personalized care in hospice settings. The variability in patient responses suggests that IPOS is effective in capturing the individual needs and concerns of patients nearing the end of life. Moreover, its multidimensional structure enables a holistic understanding of patient well-being, facilitating the creation of an Individualized Care Plan (ICP) that goes beyond the management of physical symptoms. The collaborative use of IPOS by physicians, nurses, psychologists, social workers, and volunteers helps to enhance quality of life through interdisciplinary coordination and patient-centered care.

## Data Availability

The datasets generated and analyzed during the current study are not publicly available to protect participant confidentiality but are available from the corresponding author on reasonable request and with the participants’ consent.

## Table 1 Long description

The table summarizes participant demographics, clinical characteristics, awareness levels, and hospital stay durations. Age ranged from 53 to 93 years, with an average of 75.37 years. Gender was 63 percent male (24) and 37 percent female (14), and nationality was mainly Italian at 87 percent (33). Most participants had carcinoma at 89.5 percent (34), while myeloma, leukemia, lymphoma, and other pathologies were each 3 percent (1). Psychiatric pathology was reported as yes for 16 percent (6) and no for 84 percent (32). Hospitalization days increased across time points: average 5.39 days at Time 1, 11.23 days at Time 2, and 24.05 days at death, with ranges widening from 2 to 17 up to 4 to 67. Awareness of diagnosis was mostly high at 63 percent (24), but awareness of prognosis was mostly low at 66 percent (25), indicating prognosis understanding lagged behind diagnosis understanding. Percentages may not sum to exactly 100 due to rounding.

Navigate back to Table 1.

## Table 2 Long description

The table reports, for each IPOS item at time 1, how many participants fell into low, moderate, or high symptom levels, and how each item related to awareness of diagnosis, prognosis, and overall awareness in a sample of 38. Most physical symptoms were rated low by a majority, including pain, nausea, and vomiting, while constipation and oral problems most often fell in the high range among those with data. Weakness and mobilization problems were more mixed, with substantial shares in the high range. In the psychological domain, family anxiety was frequently high, and patient anxiety was split between low and high. The clearest relationships with awareness were negative links between prognosis awareness and patient anxiety, and between prognosis awareness and reduced peace; reduced peace also showed a negative link with total awareness. Most other correlations with diagnosis, prognosis, or total awareness were small and not marked as statistically reliable, and some items had fewer respondents than the full sample.

Navigate back to Table 2.

## Table 3 Long description

The table compares IPOS item scores at two time points for 26 participants, reporting mean with standard deviation and a Wilcoxon test for change over time. In the psychological domain, anxiety rose from 1.52 (1.56) at Time 1 to 2.08 (1.69) at Time 2, with a statistically significant change (p .044). Depressive feeling increased from 1.08 (1.50) to 1.54 (1.61) but did not meet the significance threshold (p .054), and family anxiety increased from 2.68 (1.21) to 2.96 (1.02) without significance (p .083). Physical symptoms showed small, non-significant shifts, such as weakness increasing from 1.73 (1.48) to 2.00 (1.65), constipation increasing from 2.85 (1.25) to 3.08 (1.29), and drowsiness increasing from 1.77 (1.56) to 2.15 (1.40). Several physical items changed little or decreased slightly, including nausea from 0.50 (1.21) to 0.31 (0.88) and shortness of breath from 0.85 (1.16) to 0.81 (1.33). Spiritual and relational items also showed no significant changes, including feeling at peace (reverse scored) from 0.36 (0.81) to 0.31 (0.93) and practical problems from 0.75 (1.25) to 1.12 (1.51). Overall, the main finding is a significant increase in anxiety, while other observed differences should be interpreted cautiously because their p values did not indicate statistical significance.

Navigate back to Table 3.
